# Influence of an increased content of pea and yellow lupin protein in the diet of pigs on meat quality

**DOI:** 10.1186/s40813-021-00242-x

**Published:** 2021-12-19

**Authors:** Aleksandra Cebulska, Hanna Jankowiak, Eva Weisbauerová, Pavel Nevrkla

**Affiliations:** 1 Department of Animal Breeding and Nutrition, Faculty of Animal Breeding and Biology, UTP-University of Science and Technology in Bydgoszcz, Mazowiecka 28, 85-084 Bydgoszcz, Poland; 2 Institute of Animal Science, Prague-Uhříněves, Komenského 1239, Kostelec nad Orlicí, 517 41 Czech Republic; 3 Departament of Animal Breeding, Faculty of AgriSciences, Mendel University in Brno, Zemědělská 1, 613 00 Brno, Czech Republic

**Keywords:** Fattening pigs, Nutrition, Soya bean extraction meal, Pea and yellow lupin, Meat quality traits

## Abstract

**Background:**

The aim of the study was to test the effect of replacing soya beans with pea and yellow lupin seeds in the diet of pigs on meat quality. The meat for the tests was obtained from 60 fattening crossbred pigs F_1_ (Polish Large White × Polish Landrace)  × F1 (Pietrain × Duroc). The animals belonged to three feeding groups depending on the feed used with the total share of soybeans and its reduction. Water holding capacity, colour, and tenderness were measured and visual and tactile evaluation (colour, marbling and firmness) was performed for meat samples collected from the *longissimus lumborum* muscle. The chemical composition of the meat and the content of minerals were determined. The content of amino acids, fatty acids and cholesterol was determined.

**Results:**

There was no significant differences among the assessed physicochemical characteristics of the meat. The obtained meat was of good quality, regardless of the proportion of proteins from legumes in the diet of pigs. The results of the subjective evaluation of meat, its colour, and the content of muscle pigments were uniform in all food groups. Similarly, a uniform, high protein content was found in all tested groups (C—24.98%; E1—24.82%; E2—25.09%) and the content of macro- and micronutrients in the tested meat was equivalent. The profile of fatty acids was not significantly affected by dietary treatment. Palmitoleic acid content was significantly higher (*P* < 0.05) in the E2 group compared to the E1 group (3.279% compared to 2.844%). The content of amino acids in meat samples was influenced by dietary treatment. Almost all the monitored essential amino acids (threonine, valine, leucine, phenylalanine and lysine) and some of nonessential amino acids’ proportion was increased in the experimental groups (E1, E2).

**Conclusion:**

Replacing soya bean protein in the pigs’ diet with legume protein (peas and yellow lupin) did not adversely affect meat quality. This applies to both the physicochemical characteristics, the basic composition and the fatty acid profile. The meat of fattening pigs fed with the highest proportion of pea and lupin in the ration was characterized by more favourable proportion of essential amino acids.

## Background

The quality of pork largely depends on the breed, but also on environmental factors such as nutrition, raising conditions and pre-slaughter handling procedures. Nutrition is one of the most significant of the environmental factors [1]. The use of certain feeds in the feeding of fattening pigs may significantly affect the physicochemical characteristics and the content of certain nutrients [2]. This also applies the content and origin of protein taken up by pigs in their feed [3].

The most commonly used protein component in complete feed mixtures for fattening pigs is soya bean extraction meal [4]. Soya bean is one of the plants that are improved by genetic modification to a great extent, which is beneficial from the point of view of cultivation and yields [5]. Unfortunately, it is related to consumers’ fear of consuming meat and other animal products derived from the use of genetically modified organisms (GMOs). Concern about the impact of products made with the use of GMO feeds on the health of consumers causes a growing reluctance to use this type of feed [6]. In addition, the desire to become independent from the import of soya bean meal, which currently amounts to approximately 2 million tonnes per year in Poland, becomes increasingly pronounced in all Europe [7, 8]. This also results in a greater interest in feedstuffs containing protein sources other than soya beans. These are legumes, such as: lupins, peas or field beans, which, compared to other legumes, are characterised by a high protein content (over 40% in lupin seeds), high energy content and a low content of fibre and anti-nutritional compounds in pea seeds [9, 10].

An important aspect of the produced livestock is obtaining good quality meat. Many features are taken into account in the qualitative assessment. The best known are: color, drip loss, tenderness [11, 12] The results of numerous studies on the effect of feeding pigs with a complete feed mixture with a different proportion of legumes, indicate that it is possible to successfully replace soya bean extraction meal with pea or lupin seeds in the diet of pigs with no adverse impact on the quality of meat [13–16].

The aim of the study was to test the effect of replacing soya beans with pea and yellow lupin seeds in the diet of pigs on meat quality.

## Results

The physicochemical characteristics of the meat were presented in Table 1. There was no significant differentiation of the assessed characteristics depending on the source of protein in the provided feed. The results obtained in all groups were equivalent. The acidity of the meat, both at the beginning (pH_1_) and 48 h after slaughter (pH_u_), and parameters of the loss of juice from the meat was characteristic for good quality meat. In the case of tenderness, there weren’t differences between the assessed groups. All the results concerning the physicochemical characteristics of the meat of fattening pigs fed with soya beans and hard-seeded legumes were typical for good quality meat.

**Table 1:** Physicochemical characteristics of meat

	Feeding group				
	C	E1	E2	SEM	*P* <
pH_45_	6.33	6.34	6.29	0.02	0.595
pH_u_	5.22	5.22	5.18	0.01	0.225
WHC (% of free water)	17.42	16.81	18.07	0.42	0.466
Thermal drip (%)	24.50	24.88	24.15	0.28	0.578
WBSF (N)	45.81	50.16	52.02	1.17	0.072
*Chemical composition of meat*					
Water content (%)	72.42	72.45	72.18	0.09	0.461
Total protein content (%)	24.98	24.82	25.09	0.08	0.439
IMF (%)	1.43	1.21	1.39	0.10	0.625

*pH*_*45*_ pH at 45 min post slaughter, *pH*_*48h*_ pH at 48 h post slaughter, *WHC* Water holding capacity, *WBSF* Warner Braztler shear force (*N* Newton), *IMF* Intramuscular fat content

The results of visual and tactile and instrumental evaluation of meat, colour and muscle pigments are presented in Table 2. The results of the research in this area were equivalent in all feeding groups. The visual and tactile evaluation of the meat colour and the awarded points indicated that the test samples were characterised by an eye-pleasing, red-pink colour. This was confirmed by the instrumental evaluation of the colour brightness and the share of red and yellow colour in the meat. These parameters, in all analysed groups, reached the values typical for good quality meat.

**Table 2:** Visual and tactile evaluation of meat, its colour and muscle pigments

	Feeding group				
	C	E1	E2	SEM	*P* <
*Visual and tactile evaluation*					
Visual colour intensity (1–6 scale)	2.8	3.1	3.1	0.07	0.137
Marbling (1–10 scale)	1.6	1.6	1.5	0.07	0.523
Firmness (1–7 scale)	4.2	4.1	4.3	0.05	0.351
*Instrumental evaluation*					
Colour measurements					
L*	56.95	56.07	56.68	0.32	0.496
a*	13.90	14.12	14.10	0.11	0.684
b*	4.50	4.41	4.61	0.13	0.827
C*	14.66	14.83	14.87	0.09	0.613
h^o^	18.03	17.39	18.17	0.58	0.849
Muscle pigments (micrograms of hematin per 1 g of meat)	35.26	35.43	35.19	1.06	0.996

L*—value represents lightness, a*—participation of red, b*—participation of yellow, C*—saturation, h^o^—hue angle

Table 3 presents data concerning the content of macro- and microelements of the assessed meat samples. There were no statistically significant differences for the given characteristics between individual feeding groups C, E1 and E2. However, it can be observed that among the macronutrients, phosphorus and potassium were the most frequent, while among the micronutrients, higher amounts of zinc and iron were recorded.

**Table 3:** Content of minerals in meat

Minerals (mg*100 g^−1^)	Feeding group				
	C	E1	E2	SEM	*P* <
P	209.79	196.34	197.05	3.83	0.264
K	393.59	392.63	389.28	4.24	0.914
Ca	7.52	6.3	7.11	0.21	0.064
Mg	27.73	27.41	27.55	0.98	0.994
Na	40.67	46.48	38.92	1.48	0.094
Fe	0.85	1.21	0.93	0.18	0.717
Zn	1.41	1.42	1.48	0.02	0.423
Cu	0.05	0.05	0.05	0.00	0.716
Mn	0.02	0.02	0.01	0.00	0.535

The content of fatty acids in meat samples is illustrated in Table 4. The profile of fatty acids was not significantly affected by dietary treatment. Palmitoleic acid content was significantly (*P* < 0.05) higher in E2 group compared to E1 group (3.279 resp. 2.844 g/100 g of total fatty acids). The proportion of n-6/n-3 polyunsaturated fatty acids was not significantly affected by the diet. The level ranged from 6221 to 6812. The atherogenic and thrombogenic indexes did not significantly differ among the groups.

**Table 4:** Fatty acid profile and dietary indices

Fatty acid (% of total FA)	Feeding group				
	C	E1	E2	SEM	*P* <
*SFA*^1^					
Lauric (C12:0)	0.424	0.421	0.386	0.029	0.839
Myristic (C14:0)	1.316	1.186	1.136	0.041	0.183
Pentadecanoic (C15:0)	0.367	0.313	0.348	0.017	0.422
Palmitic (C16:0)	18.990	19.026	19.227	0.194	0.870
Margaric (C17:0)	0.401	0.335	0.344	0.017	0.235
Stearic (C18:0)	9.535	9.753	9.887	0.146	0.616
Total SFA	44.022	43.630	43.302	0.365	0.369
*MUFA*^2^					
Palmitoleic (C16:1n-7)	3.222	2.844^a^	3.279^b^	0.070	0.019
Oleic (C18:1n-9)	33.712	34.265	35.216	0.386	0.278
C18:1n-7	3.470	3.321	3.575	0.050	0.115
Eicosenoic (C20:1n-9)	0.669	0.674	0.661	0.011	0.892
Total MUFA	43.251	42.957	44.577	0.411	0.233
*PUFA*^3^					
Linoleic (C18:2n-6)	7.731	8.784	7.573	0.241	0.081
Alpha-linolenic (C18:3n-3)	0.601	0.720	0.581	0.035	0.228
Eikosadienoic (C20:2n-6)	0.392	0.345	0.360	0.015	0.452
Eicosatrienoic (C20:3n-6)	0.335	0.311	0.344	0.018	0.477
Arachidonic (C20:4n-6)	1.358	1.314	1.514	0.046	0.182
EPA (C20:5n-3)	0.484	0.402	0.379	0.025	0.196
DHA (C22:6n-3)	0.509	0.366	0.452	0.027	0.098
Total PUFA	12.727	13.413	12.121	0.284	0.395
*Indices*					
Total PUFAn-6	10.657	11.423	10.527	0.253	0.299
Total PUFA n-3	1.930	1.798	1.757	0.075	0.619
PUFA n-6/PUFA n-3	6.352	6.812	6.221	0.305	0.714
AI^4^	0.444	0.432	0.427	0.007	0.622
TI^5^	0.768	0.764	0.777	0.011	0.893
SFA/uSFA^6^	0.786	0.774	0.764	0.012	0.446

^(a^^−^^b)^ Row means with different superscripts differ significantly at *P* < 0.05

^1^*SFA* saturated fatty acid, ^2^*MUFA* monounsaturated fatty acid, ^3^*PUFA* polyunsaturated fatty acid, ^4^*AI* atherogenic index, ^5^*TI* thrombogenic index, ^6^*uSFA* unsaturated fatty acid

The content of amino acids in meat samples was influenced by dietary treatment. The results are illustrated in Table 5. All monitored amino acids proportion was increased in experimental groups (E1 and E2), but not all significantly. Essential amino acid content, except methionine, was higher in E2 group compared to the C group (*P* < 0.05). There was not found significant difference in essential amino acid content between E1 and E2 group. The highest content was found for lysine (from 6.887 to 7.468 g/100 g). Nonessential amino acids were also affected by the diet with a different level of statistical significance. The content of serine, proline, alanine, tyrosine and cysteine was not significantly affected by the diet. Aspartic acid, glutamic acid, glycine, histidine and arginine content was significantly higher in E2 group compared to the control group (*P* < 0.5—*P* < 0.01). The difference between E1 and E2 group was monitored only in aspartic acid, glutamic acid, histidine and arginine (*P* < 0.5—*P* < 0.01). The highest content was measured for glutamic acid (from 11.633 to 12.715 g/100 g). The lowest proportion was found for cysteine; its content ranged from 1.109 to 1.188 g/100 g.

**Table 5:** Amino acid content in meat

Amino acid (g/100 g)	Feeding group				
	C	E1	E2	SEM	*P* <
*Essential*					
Threonine	3.619^a^	3.643	3.889^b^	0.044	0.023
Valine	3.907^a^	3.987	4.279^b^	0.056	0.019
Leucine	6.246^a^	6.284	6.705^b^	0.076	0.025
Isoleucine	3.645^a^	3.682	3.969^b^	0.049	0.015
Phenylalanine	3.203^a^	3.250	3.488^b^	0.047	0.033
Lysine	6.887^a^	6.904^a^	7.468^b^	0.085	0.006
Methionine	2.257	2.389	2.320	0.034	0.275
*Nonessential*					
Aspartic acid	7.177^A^	7.360^a^	7.893^Bb^	0.085	0.002
Serine	3.102	3.115	3.287	0.036	0.071
Glutamic acid	11.633^A^	11.674^A^	12.715^B^	0.140	0.001
Proline	3.171	3.358	3.308	0.071	0.536
Glycine	3.212^a^	3.285	3.445^b^	0.036	0.030
Alanine	4.352	4.357	4.617	0.051	0.059
Tyrosine	3.106	3.065	3.424	0.099	0.286
Histidine	3.482^A^	3.549^a^	3.814^Bb^	0.044	0.006
Arginine	4.902^A^	4.959^A^	5.439^B^	0.067	0.001
Cysteine	1.109	1.172	1.188	0.026	0.453

^(A^^−^^B)^ Row means with different superscripts differ significantly at *P* < 0.01

^(a^^−^^b)^ Row means with different superscripts differ significantly at *P* < 0.05

## Discussion

The demand for raw materials rich in proteins for the production of forages is very high (e.g., in Poland it is approximately 1639 thousand tonnes of raw material rich in proteins). It is mainly satisfied by imported soya bean extraction meal (approximately 64%). At the same time, attention is paid to the production of seeds of native hard-seeded legumes. The research conducted in this field indicates the possibility of replacing this protein raw material in mixed feeds without adversely affecting the efficiency of pork production and the quality of the obtained products.

The results of numerous studies on the effect of feeding fattening pigs with a feed mixture with a different proportion of legumes did not show any significant differences in the characteristics of meat quality [8, 13, 14, 17–20]. Moreover, the values of individual physicochemical characteristics indicate that the meat obtained after the application of a diet rich in seeds of legumes grown in Poland is good quality [13, 21–23]. Which was also confirmed in this study. The studies by Milczarek and Osek [22] showed a beneficial effect of replacing soya bean seeds in the diet of pigs by 10% and 20% low-tannin field beans on the content of free water (WHC) in meat, which grew by over 2%. In turn, Sońta et al. [23] demonstrated that partial replacement of soya bean extraction meal with 7.5% yellow lupin resulted in a significantly higher (by 1%) drip loss of meat. The situation regarding the loss of meat juice in this study was different, in which no statistically significant differences were noted between the groups fed with a different proportion of peas and lupines in diet. The other hand, Milczarek and Osek [24] obtained a lower drip loss in the meat of Puławy breed fattening a diet with the addition of 10% low-tannin field bean seeds than in the meat of pigs fed with a mixed feed containing soya bean extraction meal.

The research conducted in Poland and abroad, and also the results presented above, they did not show a negative impact of replacing soya beans in a complete feed mixture with legumes on the level of total protein and intramuscular fat in pork [13, 14, 23, 25, 26]. The lower content of total protein in meat (about 2%) compared to the results obtained in this study was obtained in the studies by Chrenková et al. [14], who used a mixture with a 30% addition of peas in the diet of pigs, and Zraly. et al. [20] who used a 10% addition of lupin. Mordenti et al. [10] also obtained a lower protein content (21.05%) in the meat of pigs fed the mixture with the addition of field beans. On the other hand, a higher intramuscular fat content in meat, by 1%, was obtained in the studies by Bocian et al. [13], Chrenková et al. [14], Zralỳ et al. [20]. Fiedorowicz-Szatkowska et al. [27] showed a lower content of intramuscular fat in the meat of pigs fed with the addition of peas, compared to meat obtained from fattening pigs fed with a mixed feed based on soya beans as the main source of protein. The results described above, however, were not reflected in own research, in which the content of intramuscular fat was similar in all the studied groups of pigs.

One of the important parameters for assessing the quality of meat is its colour, as it determines the preferences of consumers during its purchase. The results of visual, tactile, and instrumental evaluation of meat, its colour and muscle pigments (Table 2) are similar to those obtained by Bocian et al. [13]. These authors showed no significant differences in the assessed characteristics between the meat samples obtained from pigs fed with a mixture based on soya beans (control group), and a diet with 20% addition of peas and 37.6% addition of lupin in the first stage of fattening as well as 38.5% addition of peas and 20% addition of lupin in the second phase (experimental group). Stein et al. [28] also showed that meat obtained from pigs fed a diet with an increased content of peas (66%) was darker and with a more desirable colour (3.22 points) in comparison with pigs fed with forages containing soya bean extraction meal (2.41 points). In this study, the brightness L* of meat colour in the studied groups of pigs was at a similar level (56.07–56.95). The obtained L* values indicate meat with a lighter colour than the values indicated in the studies performed by Milczarek and Osek [22], Sońta et al. [23], Hanczakowska and Świątkiewicz [15], where in the meat of pigs fed a diet with a varied content of legumes, a darker colour (L* 49–52) was observed. Probably the light color of meat was a result of participation of Pietrain breed in hybrids used in this research. Grabež et al. [29] demonstrated that meat obtained from fattening pigs fed fodder containing rapeseed and field bean seeds as the main source of protein was characterised by a darker, more desirable colour and a much lower proportion of yellow.

The content of minerals in pork determines its nutritional value. The applied feeding of pigs with a diet with a different proportion of legumes did not adversely affect the content of minerals in the meat (Table 3). In the study by Milczarek and Osek [24] concerning the meat of Puławy breed pigs fed a mixture with 10% of low-tannin field beans, the obtained amounts of mineral compounds of P, K, Ca, Na, Zn and Mn were similar; the amount of Cu was higher (0.088–0.104 mg*100 g^−1^), while the amount of Fe was lower (0.89–1.02 mg*100 g^−1^) in comparison to the present study (Table 3). In turn, Stasiak et al. [26] analysed the meat of pigs fed a mixture with 35% addition of lupin and 5% addition of peas, and demonstrated a higher content of Mg (282, 34 mg*kg^−1^) but a lower content of K, Na and Fe (appropriately 3668.41; 329.35; 0.02 mg*kg^−1^) in comparison to those obtained in this study. Hanczakowska et al. [30] studied the possibility to replace a part of soybean in pig diet by 4 different varieties of lupine. During the fattening period they did not observe significant differences in weight gain. Meat from pigs fed a diet of lupin was poorer in n-3 PUFA than the meat from the control group. The content of palmitic and palmitoleic acid was significantly higher in the experimental groups and the content of stearic and alpha-linolenic acid was significantly lower. According Petterson [31] the feeding of lupin seeds to pigs is associated with alteration of the fatty acid profile in backfat. The author states that L. luteus contains 21% of oleic acid, 47% of linoleic acid, and the content of these fatty acids in fat corresponds with this finding. Also, Rybiński et al. [32] mentioned the lupin seed oil as a rich source of unsaturated fatty acids such as oleic acids. The n-6/n-3 PUFA ranges from 1:1.7 to 1:10.8 [33]. Zralý et al. [19] studied the use of white lupin in pig diet and its effect on pork quality. According authors, the lupin diet was characterised by higher content of oleic acid compared to control diet. The content of linoleic, linolenic acid, SFA and PUFA was lower in the experimental diet. But the results were not statistically significant. The same tendency was observed in our experiment. Prandini et al. [34] studied the effect of pea and faba bean seeds as a protein source in growing-finishing pig diets. Dietary treatment affected linoleic acid level, n-6 PUFA content, total MUFA and total PUFA content in the subcutaneous fat of pigs. Trombetta et al. [35] also found a low effect of the pea on the fatty acid profile of intramuscular fat. Písaříková and Zralý [36] and Písaříková et al. [37] marked the lupin seeds as a source of protein in pig diet which is characterised by lower concentration of lysine, methionine, cysteine, threonine, valine and tryptophan in comparison with other protein sources as soybean meal, fish meal or meat and bone meals. The lower availability of lysin was suggested as a problem of lupin seeds in pig diet [38] but in recent varieties the availability of lysine was improved [18].

## Conclusions

Replacing soya bean protein in pigs’ diet, even in 100% in the second stage of fattening, with legume protein (peas and yellow lupins) did not adversely affect meat quality. It should be emphasised that the obtained results of the physicochemical characteristics of the meat indicated its good quality. Moreover, meat contained a high content of total protein (approx. 25%) in its composition, and thus was characterised by a high nutritional value. Moreover, in the meat of pigs fed with an increased share of pea and lupine in the ration, more favourable proportion of essential amino acids was observed.

## Methods

### Animals and sampling

The experiment was carried out on meat obtained from 60 fattening crossbred pigs F_1_ (Polish Large White × Polish Landrace)  × F_1_ (Pietrain × Duroc), which originated from and were raised on the same farm, under the same environmental conditions, in accordance with the welfare requirements. The animals were marked and placed in identical pens equipped with an auto-feeder and automatic nipple drinker, allowing for constant access to water. The study did not require the approval of the ethics committee. They were part of the production cycle and their main purpose was to assess the quality of meat (Directive 2010/63/EU).

The animals were divided into three feeding groups, each comprising 20 animals (50% of gilts and 50% of barrows). Fattening pigs were fed ad libitum with complete feed mixtures, differing in the source of protein origin: control group C: in the 1st and 2nd fattening phase, standard nutrition with 100% of soya bean extraction meal was used; experimental group E1: in the 1st phase of fattening, the soya bean protein was replaced in 50% with the pea and lupin protein and in the 2nd phase the soya bean protein was replaced in 75% with the pea and lupin protein; experimental group E2: in the 1st phase the soya bean protein was replaced in 50% with the pea and lupin protein, and in the 2nd phase the soya bean was completely eliminated (100% pea and lupin protein). The composition and nutritional value of the complete feed mixture are provided in Tables 6 and 7.

**Table 6:** Dietary value and composition of the fodder

Dietary value	Control		E1		E2	
	Phase I (30–70 kg)	Phase II (70–115 kg)	Phase I (30–70 kg)	Phase II (70–115 kg)	Phase I (30–70 kg)	Phase II (70–115 kg)
*Composition of the fodder, %*						
Soy meal 46% total protein	16	12	10	4	10	-
Wheat 12%	20	20	20	20	20	20
Barley 12%	35	45	30	41	30	35
Triticale 10%	25.3	20	26	20	26	20
Soybean oil	1	0.3	1.3	0.3	1.3	0.3
Lupin 37%	–	–	7	9	7	12
Pea 21%	–	–	3	3	3	10
PORKOVITAL T PEA 2.5%	2.5	2.5	2.5	2.5	2.5	2.5
SELACID GG DRY 25 BR	0.2	0.2	0.2	0.2	0.2	0.2
Total	100	100	100	100	100	100
Dry matter, g	877	875	877	875	877	875
Metabolic energy, MJ	13.39	13.11	13.32	13.09	13.32	13.12
Total protein, g	170	159	171	157	171	160
Fat, g	27	20	29	20	29	20
Lysine, g	10.6	9.7	10.6	9.7	10.6	9.7
Calcium, g	5.9	5.8	5.9	5.8	5.9	5.8
Phosphorus, g	5.3	5.2	5.3	5.2	5.3	5.2
Sodium, g	1.7	1.7	1.7	1.7	1.7	1.7
Vitamin A, IU	10,000	10,000	10,000	10,000	10,000	10,000
Vitamin D, IU	2200	2200	2200	2200	2200	2200
Vitamin E, IU	80	80	80	80	80	80

**Table 7:** Composition of the concentrate

PORKOVITAL T PEA 2.5%	
*Składnik w 1 kg*	
Lizyna g	120
Metionina g	40
Treonina g	50
Tryptofan g	3
Wapń ogólny g	205
Fosfor ogólny g	80
sód g	64
Witamina E + AO-mix IU	3200
Witamina A IU	260000
D IU	80000
K mg	80
C mg	–
B1 mg	60
B2 mg	200
B6 mg	88
Niacyna mg	1200
Kwas foliowy mg	32
Pantotenian wapnia mg	640
Cholina mg	9217
Biotyna mcg	2400
Witamina B12 mcg	1600
Żelazo mg	4800
Mangan mg	2400
Cynk mg	4800
miedź mg	1000
Jod mg	44
selen mg	12

Animals mean body weight at the beginning of fattening was 31.60 ± 3.01 kg. The duration of the fattening period was 87.69 ± 7.07 days. After the fattening was complete, the animals were individually weighed and then transported to a slaughterhouse situated approximately 20 km distant. The mean body weight of fattening pigs at slaughter was 112.66 ± 2.75 kg. The animals were slaughtered in meat processing plants using the electrical stunning method.

### Meat analysis

The acidity of the meat was determined 45 min after slaughter (pH_45_) and 48 h after slaughter (pH_u_) using an pH-meter with a blade electrode (Elmetron CP 401). The device was calibrated with pH 7.0 and pH 4.0 buffers provided by Elmetron. Meat quality was assessed 48 h after slaughter on samples of the *longissimus lumborum* muscle, which were stored under refrigeration at 4–6 °C.

Water holding capacity (WHC) was determined using the method developed by Grau and Hamm [39] as modified by Pohja and Niinivaar [40]. A 300 mg sample of ground meat was applied to a Whatman 1 type filter paper, placed between two glass plates and subjected to a uniformly distributed load of 2 kg for 5 min. The percentage of free water in the meat was calculated based on the drip surface area, assuming that 1 cm^2^ of the drip corresponds to 10 mg of water [11]. The drip surface area of the meat juice was measured using the LUCIA image analysis software (System for Image Processing and Analysis, version 4.82.2004). Thermal drip was determined 48 h after slaughter using the Walczak method [41]. A sample of ground meat (20 g) was placed in hygroscopic gauze and heated in a water bath at 85 °C for 10 min. After the removal of the sample and the gas, the sample was cooled down to 4 °C and weighed. Percentage loss was calculated based on the difference in meat weight before and after heat treatment.

Tenderness was measured using the INSTRON 3342 universal testing machine with a Warner–Bratzler Shear Force (WBSF) in accordance with the methodology provided by Szalata et al. [42]. A sample of meat weighing approximately 120 g was heated in a water bath until the temperature inside the bath reached 70 °C. Heat treatment was carried out in a NaCl solution with a concentration of 0.85%. Subsequently, 10 mm × 10 mm bars were cut out in accordance with the course of the muscle fibres, and they were cut perpendicularly to their course. The results were read in the form of the maximum shear force, expressed in N.

Chemical composition of meat, i.e., the content of water, protein and fat, was determined in accordance with the Polish standard [43] by the method of near-infrared transmission (NIT) spectrometry using calibration on artificial neural networks (ANN) performed by the FoodScan (device provided by FOSS).

Visual and tactile evaluation was conducted 48 h after slaughter on a 120 g slice of raw meat. Visual and tactile evaluation of the meat was carried out by a 10-person trained team. All evaluators had 4 years of experience in evaluating pork. The following elements were determined visually in raw meat: visual colour intensity according to a 6-point scale [44]: 1 point—very light meat, 6—dark purple meat; marbling with the use of Canadian and American standards on a 10-point scale [45, 46]: 1—meat without overgrowth, 10—very high marbling score, and tactile evaluation: firmness on a 7-point scale [44]: 1—very hard, 7—very soft.

Meat colour was also measured on a slice of raw meat, 48 h after slaughter, using a Minolta CR 310 photo colorimeter (Konica Minolta, Japan) with a measuring port diameter of 50 mm. The device was standardised using a white CR310 standard plate with the following coordinates: Y = 92.80, x = 0.3175 and y = 0.3333 Colour parameters were determined in the CIE L*a*b* system (L*—lightness, a*—value representing redness, b*—represents yellowness) [47], with the use of a D65 illuminant and a 2° standard observer. Chroma (C*) and hue angle (h^o^) were calculated according to Beattie et al. [48] and Brewer et al. [49]:

$$C* = \sqrt {a*^{2} + b*^{2} } ,h^\circ = tan^{ - 1} \frac{b*}{{a*}}$$

Muscle pigments were determined colourimetrically according to the Hornsey method [50]. Ground meat samples (10 g) were covered with 40 ml of acetone: water: concentrated HCl mixture at a ratio of 40:2:1 and extracted for 1 h. After filtration, the absorbance of the test solutions was measured with a Marcel Media spectrophotometer at a wavelength of 640 nm. The optical density value (E) was multiplied by a factor of 680 to obtain the correct hematin concentration expressed as micrograms of hematin per 1 g of meat.

The content of minerals in the meat was determined by atomic absorption spectrometry (Solaar 969 device) [51, 52]. For this purpose, freeze-dried meat samples, subjected to wet mineralisation (Ethos Plus microwave mineraliser), were used.

Fatty acid composition of meat was determined after chloroform methanol extraction of total lipids [53]. Fatty acid methyl esters were prepared in accordance with CSN ISO 5509 [54] and analysed by gas chromatography (gas chromatograph 6890 N Agilent Technologies) according to CSN ISO 5508 [55]. The gas chromatograph was equipped with DB-23 cyanopropylmethylpolysiloxane column (150 to 230 °C). Fatty acids were determined by comparison with standards (37 Component FAME Mix, PUFA No. 1, PUFA No. 2, PUFA No. 3; Sigma-Aldrich). Results were expressed as percentages of the total fatty acid.

The atherogenic index (AI) and thrombogenic index (TI) were calculated in accordance with Ulbricht and Southgate [56]:

$$AI= \frac{C12:0+4C14:0+C16:0}{\sum MUFA+\sum \left(n-6\right)+\sum (n-3)}$$

$$TI= \frac{C14:0+C16:0+C18:0}{0.5\sum MUFA+0.5\sum \left(n-6\right)+3\sum \left(n-3\right)+\frac{\sum \left(n-3\right)}{\sum \left(n-6\right)}}$$

The representative samples of meat were homogenised and subjected to chemical analyses to determine selected amino acids using an Amino Acid Analyser AAA 400 (INGOS Ltd., Prague, Czech Republic, evaluation by the ChromuLan programme) equipped with an ion-exchange column. Amino acids were released from the protein molecules by acid hydrolysis with 6 M hydrochloric acid.

### Statistical analysis

The meat quality results met the assumptions of normal distribution. It was verified by the Shapiro–Wilk test. The arithmetic mean and standard deviation for the characteristics of the slaughter value and the standard error of the mean (SEM) for the meat quality characteristics were calculated. The significance of differences between C, E1 and E2 feeding groups was calculated using the Tukey’s HSD test for equal group sizes. A probability *P* < 0.05 was considered statistically significant. All calculations were performed using Statistica 13.3 PL software [57].

## Data Availability

All data generated or analysed during this study are included in this published article.
